# On the Quantification of Visual Texture Complexity

**DOI:** 10.3390/jimaging8090248

**Published:** 2022-09-10

**Authors:** Fereshteh Mirjalili, Jon Yngve Hardeberg

**Affiliations:** Department of Computer Science, Norwegian University of Science and Technology (NTNU), 2815 Gjøvik, Norway

**Keywords:** texture complexity, visual perception, first-order image descriptors, co-occurrence matrix, local binary pattern, Gabor filtering, color space

## Abstract

Complexity is one of the major attributes of the visual perception of texture. However, very little is known about how humans visually interpret texture complexity. A psychophysical experiment was conducted to visually quantify the seven texture attributes of a series of textile fabrics: *complexity*, *color variation*, *randomness*, *strongness*, *regularity*, *repetitiveness*, and *homogeneity*. It was found that the observers could discriminate between the textures with low and high complexity using some high-level visual cues such as randomness, color variation, strongness, etc. The results of principal component analysis (PCA) on the visual scores of the above attributes suggest that *complexity* and *homogeneity* could be essentially the underlying attributes of the same visual texture dimension, with *complexity* at the negative extreme and *homogeneity* at the positive extreme of this dimension. We chose to call this dimension *visual texture complexity*. Several texture measures including the first-order image statistics, co-occurrence matrix, local binary pattern, and Gabor features were computed for images of the textiles in sRGB, and four luminance-chrominance color spaces (i.e., HSV, YC_b_C_r_, Ohta’s I_1_I_2_I_3_, and CIELAB). The relationships between the visually quantified texture complexity of the textiles and the corresponding texture measures of the images were investigated. Analyzing the relationships showed that simple standard deviation of the image luminance channel had a strong correlation with the corresponding visual ratings of texture complexity in all five color spaces. Standard deviation of the energy of the image after convolving with an appropriate Gabor filter and entropy of the co-occurrence matrix, both computed for the image luminance channel, also showed high correlations with the visual data. In this comparison, sRGB, YC_b_C_r_, and HSV always outperformed the I_1_I_2_I_3_ and CIELAB color spaces. The highest correlations between the visual data and the corresponding image texture features in the luminance-chrominance color spaces were always obtained for the luminance channel of the images, and one of the two chrominance channels always performed better than the other. This result indicates that the arrangement of the image texture elements that impacts the observer’s perception of *visual texture complexity* cannot be represented properly by the chrominance channels. This must be carefully considered when choosing an image channel to quantify the *visual texture complexity*. Additionally, the good performance of the luminance channel in the five studied color spaces proves that variations in the luminance of the texture, or as one could call the *luminance contrast*, plays a crucial role in creating *visual texture complexity*.

## 1. Introduction

Visual complexity is a well-known concept in many disciplines including neuroscience [1,2], cognitive psychology [3,4,5], product marketing [6,7], web design [8,9], art [10], etc. From a theoretical point of view, studying visual complexity has revealed information about the human visual system operation when evaluating stimuli [2], the memory formation mechanism [1], and its capacity [11]. It has also helped psychologists to understand how the visual complexity of scenes impacts emotion and attracts attention [12]. From a practical perspective, research into visual complexity has aided the marketing industry in being able to better capture the consumer’s attention through pleasing and understandable advertisements [7]. It has also helped website designers to understand the influence of design features including complexity on the initial user impressions [9] and the cognitive effort a user needs to make to interact with web pages [8].

Visual complexity research has inevitably found its way into the domain of computer vision. The impact of complexity on the segmentation of histopathology images for the computer-aided diagnosis of cancer [13] and object detection in cluttered images [14,15] are two of the applications where visual complexity has played a critical role.

In the field of visual appearance, complexity might be naturally related to any of the appearance attributes, whether it be color, gloss, translucency, or texture, separately or in combination. For instance, spatio-chromatic complexity is associated with spatial and color variations on a surface that involves the perception of both color and texture at the same time [16]. The International Commission on Illumination (CIE) has assigned the technical committee “8–14: Specification of spatio-chromatic complexity” to devoting research on this visual phenomenon [17].

Research has provided significant evidence that humans are capable of perceiving and evaluating the complexity of scenes. A scene can be perceived as visually complex depending on the type and number of elements it contains and their spatial layout [18]. Various definitions for visual complexity have been proposed in the literature. The theory of complexity perception was introduced by Birkhoff [19] as an attempt to propose an aesthetic measure of an art object as the ratio between its order and complexity. He defined complexity as “a preliminary effort of attention, which is necessary for the act of perception”. In an attempt to prepare a standardized set of images for memory and cognitive processing research, Snodgrass and Vanderwart [20] found that familiarity and visual complexity are two independent attributes of a set of 260 line-drawn images. They defined visual complexity as “the amount of detail or intricacy” in the image. Heaps and Handel [21] defined visual complexity of the natural texture images as “how difficult it would be to give a verbal description of the texture”.

Complexity has been proven to be one of the main attributes of texture perception by several research works [22,23,24,25,26,27,28]. In one of the earliest studies on the visual properties of texture, Amadasun and King [22] introduced coarseness, contrast, busyness, complexity, and texture strength as the five properties of texture. They defined the texture complexity as the visual content of a texture, where the visual content referred to the patches or primitives, lines, and/or sharp edges present in the texture, having different intensities.

In an attempt to find the texture features that are important in texture perception, Rao and Lohse [23] asked twenty participants to perform an unsupervised classification of thirty pictures from Brodatz’s album [29], according to their similarity in texture. The results of multidimensional scaling analysis (MDS) indicated that perceptual texture can be characterized by the aid of only three features (i.e., repetition, orientation, and complexity). In a similar work, Mojsilovic et al. [24] performed a similarity analysis followed by multidimensional scaling (MDS) and hierarchical cluster analysis on a series of twenty color patterns from an interior design catalog. The main goal of the work was to find the basic dimensions of color patterns and their relationships. As a result, five categories that observers used to judge the similarity and matching of color patterns were found: overall color, directionality and orientation, regularity and placement, color purity, and complexity and heaviness. The fifth dimension, pattern complexity, and heaviness seemed to contain both the perceived color and luminance information.

Guo et al. [25] investigated the human *Kansei* of the complexity of visual scenes with a focus on texture perception. Kansei, a Japanese-origin word, is a technical term widely used in industrial design. Kansei engineering is a method of translating the consumer’s psychological needs and feelings for a product into design elements [30]. Through the verbal description of the texture of twenty images from Brodatz’s album and a subsequent factor analysis, they found that the major characteristics of texture that affect the human perception of visual complexity are regularity, roughness, understandability, density, and directionality. Giocca et al. [26,27] studied the complexity perception of fifty-four natural color images from the VisTex texture dataset [31]. The images were shown to seventeen participants on a web-interface where they rated the complexity of the images on a scale of 0–100 and verbally described the characteristics of textures that impacted their evaluation of complexity. Regularity, understandability, edge density, and familiarity were found to be the main criteria they used to evaluate complexity.

Some studies have addressed the possibility of quantifying the texture complexity. Giocca et al. [27] compared the Haralick features [32], also known as the gray level co-occurrence matrix (GLCM) features, and the frequency factor, edge density, colorfulness, color harmony, etc., as potential complexity measures for the chosen VisTex images. None of the studied features showed strong correlations with the corresponding subjective complexity ratings of the images.

Ivanovici et al. [33] studied the notion of complexity in the color texture domain. They introduced a naïve complexity measure defined as three times the number of colors of the image divided by the image resolution. They compared the performance of the proposed complexity measure with the conventional color entropy computed for a series of synthetic fractal images with known complexities and with natural images of the VisTex dataset. The results revealed that the proposed naïve complexity measure correlated well with color entropy, however, both measures were only able to partially estimate the complexity of highly complex images.

In one of their recent works, Nicolae and Ivanovici [34] investigated the complexity perception of natural texture images from the VisTex dataset [31] and synthetic fractal images. In addition to a subjective complexity scoring by eight young participants, electroencephalography (EEG) was conducted to record the participants’ brain signals while observing the images. They did not provide a “formal” definition of complexity to the participants. The EEG analysis indicated “uncertainly” and “indecisiveness” involved in evaluating the images with a high complexity level. They chose color entropy (CE) and color fractal dimension (CFD) as computational texture measures. While there was little agreement between the participants, the complexity scores correlated better to CFD compared to CE.

Recently, machine learning and deep learning techniques have been employed to predict the human perception of complexity. Fernandez-Lozano et al. [35] used a dataset of 800 visual stimuli divided into five categories. Each stimulus was described by 329 features based on edge detection, compression error, and Zipf’s law. The complexity of the images was evaluated by 240 observers on a scale of 1–5, with 5 representing a very complex image. They compared various machine learning regression algorithms to predict complexity, and gained an average correlation of 0.71 between the best model’s prediction of complexity with 22 features and the respective observers’ ratings.

Despite all such efforts, developing accurate computational models of visual complexity is still a demanding task. In our previous work [36], the most important perceptual attributes that observers used to interpret and evaluate texture were accumulated through the verbal description of the texture of fifty-two textile fabrics by a group of participants. *Smoothness*, *softness*, *homogeneity*, *geometric variation*, *randomness*, *repetitiveness*, *regularity*, *color variation*, *strongness*, and *complexity* were ten of the most frequently used attributes to describe the texture of the textiles. Amongst all of these attributes, *homogeneity*, *randomness*, *repetitiveness*, *regularity*, *color variation*, *strongness*, and *complexity* were evaluated merely visually by the observers. Based on the results of [36] and further analysis of the data in the present study, we will introduce and define a new texture dimension called *visual texture complexity*. We will show how *visual texture complexity* is influenced by other visual texture attributes, particularly *randomness*, *strongness*, and *color variation*. The main objectives of the present study are as follows:

- To investigate *visual texture complexity* and establish the degree to which it is correlated with *homogeneity*, *randomness*, *repetitiveness*, *regularity*, *color variation*, and *strongness* to better understand its perception. *Visual texture complexity* will be introduced and defined in detail in Section 4.1.
- To investigate the possible relationships between *visual texture complexity* and some of the most popular and effective image texture measures.
- To find the most suitable color space/s for the quantitative evaluation of *visual texture complexity*.

To this end, a number of first and second-order image statistics as well as the local binary pattern and Gabor features computed in different color spaces were used as potential measures for texture *complexity*, and possible existing correlations between such measures and visual ratings of texture *complexity* were sought. Section 2 of the paper introduces the detailed description of the texture measures used in this research. The remainder of the paper is structured as follows. Section 3 describes the research method implemented to perform the psychophysical experiments and the procedure to compute the texture measures of the textile images. In Section 4, the newly defined texture dimension of *visual texture complexity* is introduced and defined, and the relationships between the visual ratings of this attribute and the corresponding texture measures are presented. Section 5 discusses our results, and Section 6 presents our concluding remarks.

## 2. Quantitative Color Texture

Although there seems to be no comprehensive definition for texture in the literature, an *image texture* has been described as the spatial arrangement of colors or luminance elements in the image, which creates visual non-uniformity [37]. Fortunately, texture analysis techniques have made provisions for characterizing the texture of color images in terms of various texture measures [38]. Analyzing the texture of color images can be performed through different approaches: pure color analysis using color descriptors [39,40], grayscale texture analysis using texture features [41], and joint approaches that combine color and grayscale texture information [42,43]. Our preliminary experiments indicated that integrative co-occurrence matrix (CoM) features [43] and opponent Gabor features [44] as joint color-texture features, which combine information across different image channels, give rise to unsatisfactory results (i.e., weak correlations with the visual ratings of complexity). Therefore, we do not provide the details of these features in this section. In the following subsections, we provide our readers with a thorough description of the meaning and mathematical background behind some of the most popular image statistics and texture features we chose to use in this research. These include the first-order image statistics, co-occurrence matrix features as the second-order image statistics as well as the local binary pattern and Gabor features. We chose these texture features because they are simple and computationally cheap and have been shown to be efficient in texture classification tasks [44].

### 2.1. First-Order Statistical Texture Features—Image Descriptors

The statistical approach for texture analysis introduces the first-order statistics of color images [45]. Such image descriptors are the simplest features for characterizing textures and refer to statistical parameters such as the mean, standard deviation, and central moments. They are computed either directly from the image pixel values or from the image histogram. The former will be, hereafter, referred to as the *global image descriptors* while the latter will be referred to as the *histogram-based image descriptors*. Global image descriptors for a three-channel color image (e.g., an sRGB image) are the mean and standard deviation of the pixel values of each image channel.

The *mean* (*µ*) is the average of image pixel values and indicative of the general brightness of the image: the higher the *µ*, the brighter the image. The *standard deviation* (*σ*) is the measure of the spread in the pixel values around *µ* [46]. A higher *σ* indicates an image with higher contrast [45]. Here, the term “contrast” refers to the difference between the minimum and maximum pixel values, and not the more complex concept of perceptual image contrast [47]. Again, it must be noted that *µ* and *σ* are calculated as global image descriptors directly from the pixel values of each image channel.

As the histogram-based image descriptors, we chose skewness or the third central moment (*m*_3_), kurtosis or the fourth central moment (*m*_4_), energy (*Enrg*_H_), and entropy (*Entp*_H_). The *skewness* (*m*_3_) and *kurtosis* (*m*_4_) show how much the histogram is deviated from a normal distribution. The *energy* (*Enrg*_H_) indicates how pixel values are distributed in the image. A higher *Enrg*_H_ means that the number of pixel values in the image is few, and the distribution is concentrated in only a small number of pixel values [45]. The *entropy* (*Entp*_H_) is a measure of how random the distribution of pixel values in the image is. This measure tends to vary inversely with *Enrg*_H_ and has occasionally been considered as a measure of complexity in the texture analysis. One of the most commonly used definitions of entropy is Shannon’s entropy [33].

The first-order image descriptors are simple and straightforward, and invariant to the image spatial translation and rotation. However, they are slightly dependent on the viewing angle, sensitive to illumination change, and reliable merely under a constant illumination condition [40].

### 2.2. Second-Order Statistical Texture Features—Co-Occurrence Matrix (CoM) Features

**Figure 1:**
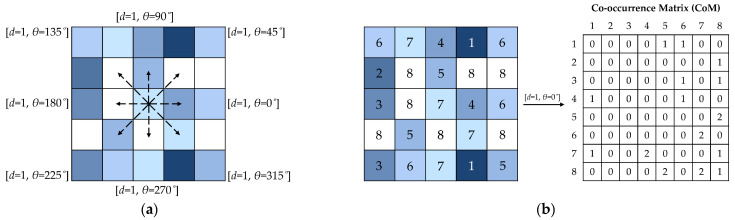
The displacement rules for generating CoMs with the distance *d* = 1 pixel, and rotation angles *θ* ∈ {0, 45, 90, 135, 180, 225, 270, 315}° (**a**). An example of a CoM for [*d* = 1, *θ* = 0°] after reducing the image bit depth to *n* = 8 through quantization (**b**).

The first-order statistics provide information about the distribution of the image pixel values. However, they are unable to provide any information about the relative positions of such pixels in the image. Examining the spatial relationship between pairs of pixels across the image can be conducted through the second-order statistical features. Co-occurrence matrix (CoM) is one of the most well-known second-order statistical operators for texture analysis [32]. A CoM is a two-dimensional matrix comprising the probabilities of occurring pairs of pixels with specific pixel values at a particular displacement of distance *d* and rotation angle *θ*. Figure 1a illustrates the displacement rules to generate CoMs with the distance *d* = 1 pixe, and equally-spaced rotation angles *θ* ∈ {0, 45, 90, 135, 180, 225, 270, 315}°. An example of a CoM for [*d* = 1, *θ* = 0°], after reducing the image bit depth to *n* = 8 through quantization, is depicted in Figure 1b.

A number of statistics can be derived from the CoM. Haralick et al. [32] proposed fourteen simple and rather intuitive CoM features for texture classification. However, it has been demonstrated that some of these features are highly correlated and only five of them (i.e., energy, contrast, correlation, entropy, and homogeneity could be sufficient for texture analysis purposes) [42].

The CoM *energy* (*Enrg*_CoM_), also known as the angular second moment, indicates how uniformly pixel values are distributed in the image. The CoM *contrast* (*Cont*_CoM_) is a measure of local intensity variations in image pixel values. The CoM *correlation* (*Corr*_CoM_) is a measure of linear dependency between the pixel values at specific positions in the image. The CoM *entropy* (*Entp*_CoM_) measures the extent to which pixel values are randomly distributed in the image. The CoM *homogeneity* (*Homg*_CoM_), also known as the inverse difference moment, measures the homogeneity of the image [48].

CoMs are rather easy to implement and have proven to be very efficient in various texture analysis tasks [49,50,51,52]. Although CoM features could be made invariant to rotation and scaling, they are highly dependent on illumination: changing illumination condition results in different image pixel values, and it affects the resulting CoM [53]. To address this limitation, the local binary pattern (LBP) operator was developed as a “grayscale-invariant” texture analysis tool [54].

### 2.3. Local Binary Pattern (LBP) Features

**Figure 2:**
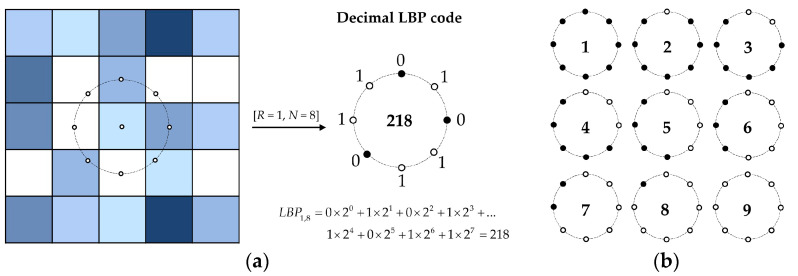
An example of generating the decimal LBP code for a pixel using *N* = 8 neighboring pixels at the radius *R* = 1 pixel: solid and hollow markers correspond to the pixels with higher and lower values as compared to the central pixel (**a**). The nine uniform binary patterns used to compute the uniform LBP features using *N* = 8 neighboring pixels as proposed in [54] (**b**).

Local binary pattern (LBP) is a powerful grayscale and rotation-invariant texture operator that has found widespread applications in texture analysis and pattern recognition due to its simplicity and discriminative efficiency [55,56]. For each image pixel, the LBP is derived from the signs of the differences between the value of that pixel, and the values of the *N* neighboring pixels surrounding it at the radius *R*. Figure 2a illustrates an example of generating the decimal LBP code for a pixel using its *N* = 8 neighboring pixels at the radius *R* = 1 pixel. Using the signs of the differences in the pixel values instead of their absolute values makes the LBP invariant to any monotonic changes in the image pixel values.

For a neighborhood of *N* pixels, the LBP operator produces 2*^N^* different binary patterns. Ojala et al. [54] observed that amongst all of the possible 2*^N^* binary patterns, only *N* + 1 patterns are associated with the most fundamental micro-structures of the texture such as bright points, dark points, edges, and corners. These patterns are known as *uniform* LBPs. Figure 2b shows the nine possible uniform binary patterns when *N* = 8, as proposed in [54]. The remaining patterns are categorized as non-uniform ones. The frequencies of the occurrence of uniform and non-uniform patterns in the image texture are recorded as a feature vector and represented as the LBP histogram.

### 2.4. Gabor Filtering

**Figure 3:**
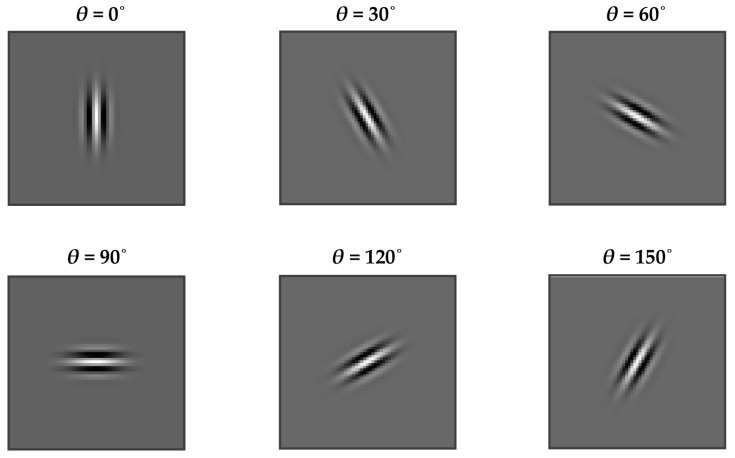
Visualizations of the real part of the Gabor function with the frequency *F* = 0.18 and orientation angles *θ* ∈ {0, 30, 60, 90, 120, 150}°.

Frequency is an essential property of many textures. Gabor filtering provides means for analyzing the image texture by decomposing it into components of varying spatial frequency (*F*) and orientation (*θ*). A Gabor filter in the spatial domain is obtained by modulating a two-dimensional complex sinusoid carrier with a two-dimensional Gaussian envelope [57]. Figure 3 shows the visualizations of the real part of the Gabor function with the frequency *F* = 0.18 and orientation angles *θ* ∈ {0, 30, 60, 90, 120, 150}°, which were used to extract the Gabor features of the image textures in this research.

The crucial step in implementing Gabor filtering for texture analysis is designing the Gabor filter bank. A filter bank comprises a number of Gabor filters with different spatial frequencies and orientations, obtained by presetting the filter parameters. After convolving the image texture by the filter bank, the mean and standard deviation of the energy of the filtered image is used as the Gabor texture features [57]. The Gabor filtering technique has been found to be very efficient in various texture analysis tasks. However, it suffers from being sensitive to changes in illumination as well as being computationally intensive [58].

## 3. Materials and Methods

### 3.1. Visual Assessment of Texture of the Textiles

As discussed in our previous work [36], the results of a verbal description experiment using 52 textile fabrics revealed that observers tend to use some essential tactile and visual cues to evaluate the textural appearance of the textiles. These cues were *smoothness*, *softness*, *homogeneity*, *geometric variation*, *randomness*, *repetitiveness*, *regularity*, *color variation*, *strongness*, and *complexity*. Based on the observers’ statements, we found that *smoothness*, *softness,* and *geometric variation* were mainly evaluated by touch, while the rest of the attributes were evaluated merely visually.

**Figure 4:**
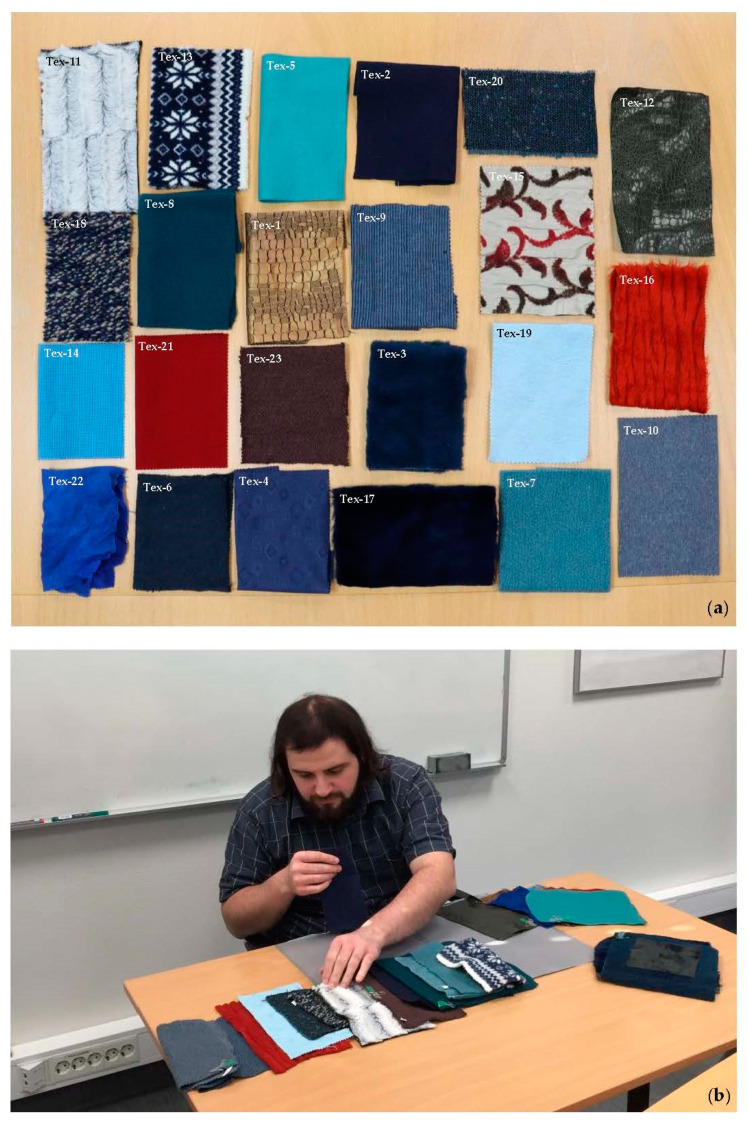
Twenty-three textile fabrics used in the rank ordering experiments. Although the sample sizes varied, we made sure that the samples were neither too small nor too large, and each sample enclosed all the essential texture primitives (**a**). An observer performed the rank ordering experiment, sorting the samples with respect to their perceived texture attribute. This image is reprinted from [36] with permission from IS&T (**b**). It must be noted that these photographs were taken with a DCLR camera and had undergone JPEG compression for the purpose of showcasing the physical samples versus their digital counterparts. This is why the appearance of the physical samples might seem different from their digital counterparts, which were rendered under standard D65 illumination. The sRGB renderings of the samples are introduced in Section 3.2 and Figure 5.

To build a foundation upon the results of [36] for more in-depth research on texture perception, we used the same textile samples in the present study. Having determined the most tangible visual attributes of texture appearance of the textiles, the next step was to quantify these attributes. The rank ordering method [59] was employed for this purpose. It was found during the verbal description experiment that the main color of the textile sample had no significant impact on its texture evaluation by the observers. For instance, the observers would describe the texture of a fur in blue the same way as they would describe the texture of that fur in red. Therefore, a subset of 23 fabrics with more or less the same main color, but different textures was selected from the sample set for the rank ordering experiments. A photograph of the selected fabrics is depicted in Figure 4.

Ten observers including seven males and three females, aging between 24 to 36 years old, with normal to corrected to normal color vision (pre-tested by the Ishihara’s test for color deficiency) participated in the rank ordering experiments.

The observers were given ample opportunity to understand the purpose of the research and their right to withdraw from the experiment before they gave their informed consent to participate. None of the observers were experts in the field of texture appearance. The rank ordering experiments were conducted in a meeting room with large windows providing natural daylight illumination. The room was also equipped with daylight fluorescent tubes. The observers sat at a table that was partially covered with a medium gray paperboard to provide a neutral background on the working area. The viewing geometry (i.e., the angle between the observer’s viewing direction and the surface normal of the table) was approximately 45°. The viewing distance (i.e., the distance between the observer’s eyes and the surface of the table-where the samples were placed) was 40 cm. The combined natural and artificial illumination on the working area had the correlated color temperature (CCT) and illuminance of approximately 6000 K and 500 lx, respectively.

After introducing written instructions about the experiment, the observers were presented with the 23 textiles. Although none of the observers was a native English speaker, they could understand and speak the English language very well. Therefore, the instruction was given in English. However, if they were unfamiliar with the meaning of an attribute, they were allowed to use online dictionaries to find the meaning. The written instructions on the experiment is given in Appendix A.

The observers had the task of ranking the textiles on the table, according to how strongly they represented the seven visual texture attributes (i.e., *complexity*, *homogeneity*, *randomness*, *repetitiveness*, *regularity*, *color variation*, and *strongness*) from the lowest to the highest of each attribute. The observers were intentionally not provided with an explicit definition of the texture or texture attributes. In fact, they were expected to use their own knowledge and understanding of texture. They were allowed to freely touch the sample, however, they had to maintain the viewing distance throughout the experiment. Since the experiment was not a forced-choice task, the observers could assign the same rank order to several samples, if needed. Figure 4 also shows an observer performing the rank ordering experiment. Each observer completed the task without a time restriction, although they would tend to finish the task in approximately one hour.

### 3.2. Image Acquisition Procedure

As mentioned previously, one of the primary objectives of this study was to investigate the possible relationships between the texture complexity and the most popular and effective image texture measures. To compute such texture measures, we acquired the sRGB images of the selected 23 textiles. The textile fabrics used in this study were part of the “Textile” class used by the HyTexila texture database [60]. The hyperspectral images of the textiles were acquired using a HySpex VNIR-1800 hyperspectral camera (Norsk Elektro Optikk AS, Oslo, Norway). The acquisition setup and procedure are fully described in [60]. Each textile was acquired by the camera to obtain a radiance image for 186 spectral bands.

The radiance images were converted to the reflectance images using the estimated spectral power distribution of the camera’s illumination system. Finally, the reflectance images were converted to sRGB images for the CIE standard illuminant D65 and CIE 1931 color matching function and cropped into images of 1024 × 1024 pixels.

**Figure 5:**
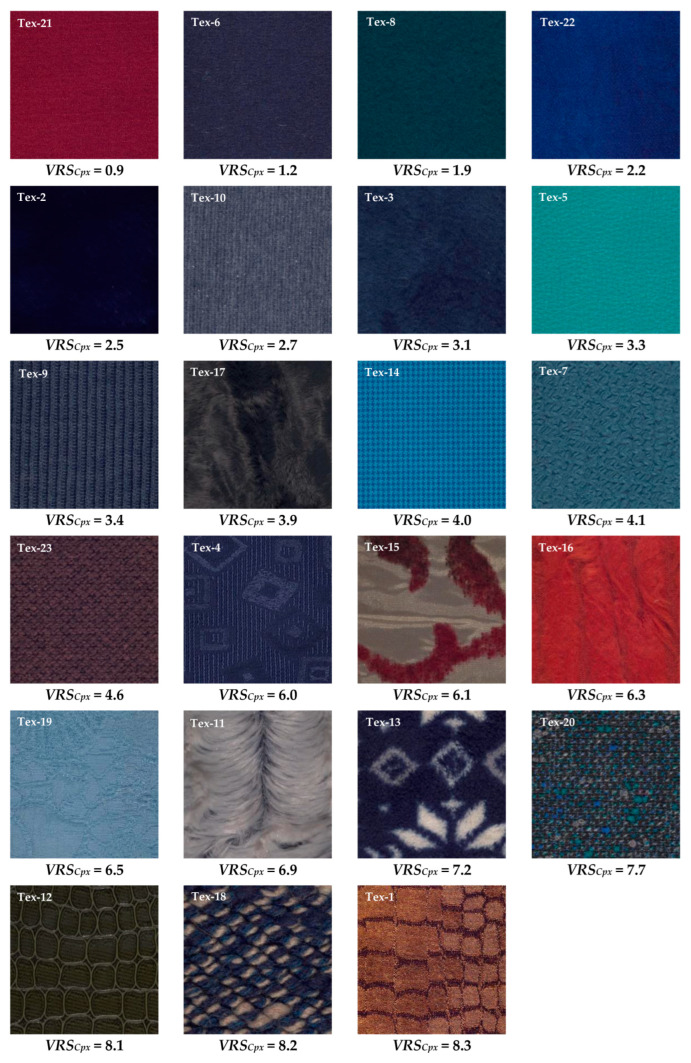
The sRGB images of the 23 textiles used in this research. The images are presented in ascending order in terms of their texture *complexity*, according to the visual rank scores (*VRS*) given by the observers. *VRS_Cpx_* refers to the *VRS* of *complexity*. Section 4.1 provides detailed information on how the *VRS* values were computed.

For our computations of image texture to be reproducible by other researchers, we performed the color calculations using D65, which was the closest CIE standard illuminant to our illumination condition in the experiment room. To ensure that the color-difference caused by the difference in illumination condition was perceptually insignificant, we measured the average CIELAB 1976 color-difference of the two conditions on the 24 color patches of the X-Rite color checker [61]. The average CIELAB 1976 color-difference was 0.86, which is below the just noticeable difference (JND) threshold [62]. This indicates a negligible perceptual difference between the two illumination conditions. Figure 5 shows the sRGB images of the textiles used to compute the texture measures. It must be noted that the textiles in Figure 5 were sorted in ascending order in terms of texture *complexity* according to their visual rank score (*VRS*) given by the observers. Section 4.1 provides detailed information on how the *VRS* was computed.

### 3.3. Computing the Image Descriptors and Texture Features of the Textile Images

Although the output of the majority of image capturing devices is color images in terms of red, green, and blue signals in the so-called RGB space, with the variety of standardized color spaces available, the question of which color space is more effective for a particular texture analysis task always remains [63]. To find the best representative color space/s for computing the image descriptors and texture features of the textile images, we chose the five popular color spaces in computer vision [64] (i.e., sRGB, HSV, YC_b_C_r_, Ohta’s I_1_I_2_I_3_, and CIELAB). They have also been used in several texture analysis and classification studies and have shown a good performance amongst the many color spaces available [65,66,67]. We followed a similar principle to find the most suitable color space/s for our application.

One of the advantages of the HSV, YC_b_C_r_, I_1_I_2_I_3_, and CIELAB color spaces compared to sRGB is that they allow for separate treatment of the luminance and chrominance components of the image [68]. The formulas used to transform the sRGB images of the textiles to HSV, YC_b_C_r_, I_1_I_2_I_3_, and CIELAB color spaces are given in Appendix B.

The six first-order image descriptors (i.e., global *µ* and *σ*), together with histogram-based *m*_3_, *m*_4_, *Enrg*_H_ and *Entp*_H_, were computed for each channel of the textile images in the sRGB space. Additionally, global *µ* and *σ* were computed for each channel of the images in the HSV, YC_b_C_r_, I_1_I_2_I_3_, and CIELAB color spaces. To compute the CoM features, the CoMs were generated for nine distances *d* ∈ {1, 5, 10, 15, 25, 50, 100, 150, 250} pixel/s and eight orientation angles *θ* ∈ {0, 45, 90, 135, 180, 225, 270, 315}° for each channel of the textile images in the five color spaces. The energy, contrast, correlation, entropy, and homogeneity of each CoM were then calculated. To obtain the rotation-invariant CoM features, the calculated features were averaged over the eight orientation angles.

Similarly, the LBP histograms were generated for eight neighboring pixels, *N* = 8, and nine radii *R* ∈ {1, 5, 10, 15, 25, 50, 100, 150, 250} pixel/s for each channel of the textile images in the five color spaces. A preliminary correlation analysis indicated that increasing *N* along with *R* had no significant impact on the results. Therefore, all LBP computations were performed with *N* = 8. For *N* = 8, the LBP histogram had ten bins, where the first nine bins were associated with the nine uniform LBPs (see Figure 2b) and the tenth bin was associated with the combination of all possible non-uniform LBPs. The histogram frequencies were used as the LBP features.

For the Gabor features, a bank of 36 Gabor filters were designed using six frequencies (*n_F_ =* 6) (i.e., *F* ∈ {1, 0.7, 0.5, 0.35, 0.25, 0.18} with the maximum frequency *F_m_* = 1) and six orientation angles (*n_O_* = 6) i.e., *θ* ∈ {0, 30, 60, 90, 120, 150}° (see Figure 3). As recommended in [34], the frequency ratio *F_r_* was half-octave frequency spacing (*F_r_* =$\sqrt{2}$) and the smoothing parameters were *γ* = *η* = 0.5. The mean and standard deviation of the energy of the filtered images were averaged over the six orientation angles to obtain the rotation-invariant Gabor features of the textile images.

## 4. Results

The outcome of the visual assessment experiments was first used to compute the visual rank scores (*VRS*) of the seven texture attributes of the textiles, and subsequently their relationships. Section 4.1 describes how *VRS* was computed and used to define *visual texture complexity*. Section 4.2 presents the results of investigating the relationships between the *VRS* of the complexity of the textiles, and the corresponding image descriptors and texture features (as introduced in Section 2) computed for the textile images in the sRGB, HSV, YC_b_C_r_, I_1_I_2_I_3_, and CIELAB color spaces.

### 4.1. Visual Texture Complexity

**Figure 6:**
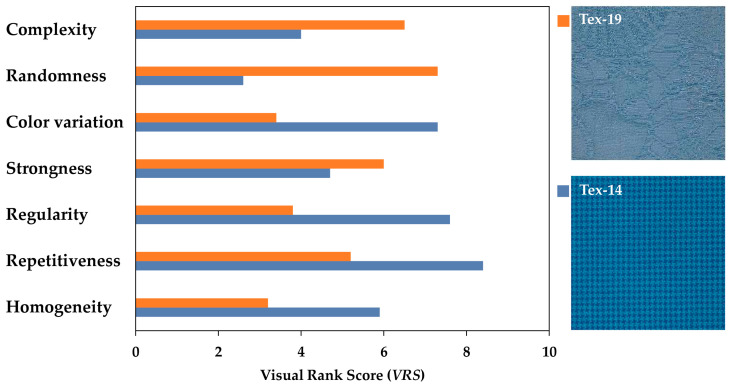
The visual rank scores (*VRS*) of Tex-19 and Tex-14 for the various texture attributes. For instance, Tex-19 had higher *complexity*, *randomness* and *strongness*, while Tex-14 had higher *color variation*, *regularity*, *repetitiveness*, and *homogeneity*.

The order of ranking of each textile was recorded for each observer, and first used to determine the accuracy of the observers in their assessments. To evaluate the extent of the observers’ accuracy in terms of the intra-observer and inter-observer variability, the standardized residual sum of squares (STRESS) metric [69] was used. The percent STRESS values were always between 0 and 100, where values of STRESS near zero indicate less variability. For intra-observer variability, the average STRESS value of the ten observers was 24 STRESS units. This value indicates that all observers were reasonably internally consistent. The average inter-observer variability of the ten observers was 38 STRESS units, which was larger than the average intra-observer variability (i.e., 24 STRESS units), as might be expected. This value indicates a reasonable degree of consistency between the observers. The visual rank score (*VRS*) of each textile was then calculated separately for each texture attribute using Equation (1) [59], and linearly re-scaled to be within the range of 0–10 for better visualization:

(1) $$VRS=\frac{1}{S(T-1)}\sum_{t=1}^{T}tQ_{t}$$

where *S* is the number of observers; *T* is the number of textile samples; and *Q_t_* is the number of times the textile *t* is ranked at position *t*. Figure 6 compares two of the textiles (i.e., Tex-19 and Tex-14 )in terms of their *VRS* computed for the seven texture attributes.

The *VRS* of *complexity*, *randomness*, *color variation*, *strongness*, *regularity*, *repetitiveness* and *homogeneity* will be, hereafter, denoted as *VRS_Cpx_*, *VRS_R_*, *VRS_CV_*, *VRS_S_*, *VRS_Rg_*, *VRS_Rp_*, and *VRS_H_* for brevity. The sRGB images of the textiles, sorted according to their *VRS_Cpx_*, from the lowest to the highest *VRS_Cpx_*, are depicted in Figure 5. A comparison of the *VRS_Cpx_* values in Figure 5 indicates that the textiles with a homogeneous structure were judged as less complex, while those with a more random structure and stronger embedded details were considered as more complex by the observers.

**Figure 7:**
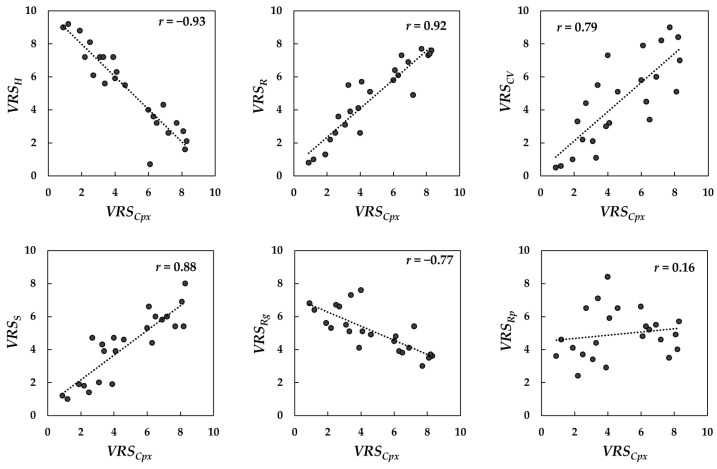
The correlation between the *VRS* of *complexity* (*VRS_Cpx_*) and the *VRS* of *homogeneity* (*VRS_H_*), *randomness* (*VRS_R_*), *color variation* (*VRS_CV_*), *strongness* (*VRS_S_*), *regularity* (*VRS_Rg_*), and *repetitiveness* (*VRS_Rp_*) for the 23 textiles used in this research.

In order to find out how the texture *complexity* is related to the other visual texture attributes, the correlations between *VRS_Cpx_* and *VRS_R_*, *VRS_CV_*, *VRS_S_*, *VRS_Rg_*, *VRS_Rp_*, and *VRS_H_* were determined in terms of the correlation coefficient (*r*) for the 95% confidence interval (*p* < 0.5). Scatter plots of such correlations are depicted in Figure 7.

Analyzing the correlations in Figure 7 showed that there were positive linear correlations between *VRS_Cpx_* and the corresponding *VRS_R_* (*r* = 0.92), *VRS_S_* (*r* = 0.88), and *VRS_CV_* (*r* = 0.79). Additionally, there were negative correlations between *VRS_Cpx_* and the corresponding *VRS_H_* (*r* = −0.93) and *VRS_Rg_* (*r* = −0.77). The correlation coefficient (*r*) for *repetitiveness* was positive but significantly low (due to the fact that there are not many samples with a repetitive texture in the sample set used). Such correlations indicate that when the texture is *random* or *strong*, or has a high content of color non-uniformity, it could be perceived as *complex*. On the other hand, when the texture is *homogenous* or *regular*, it could be perceived as less *complex*. To find the possible underlying relations between these attributes, principal component analysis (PCA) [70] was conducted on the *VRS* values. It was found that the first two principal components with eigenvalues of 31.3 and 8.4, respectively, correspond to 90% of the data variance. These coefficients are depicted in Table 1.

Comparing the PCs in Table 1 showed that PC1 had the strongest negative and positive associations with *complexity* (PC1 = −0.42) and *homogeneity* (PC1 = 0.44), respectively. The PC1, however, was much smaller for *repetitiveness* (PC1 = −0.08) and *regularity* (PC1 = 0.16), indicating that the two attributes did not correlate with PC1. On the other hand, PC2 had the strongest positive associations with *repetitiveness* (PC2 = 0.47) and *regularity* (PC2 = 0.46).

**Figure 8:**
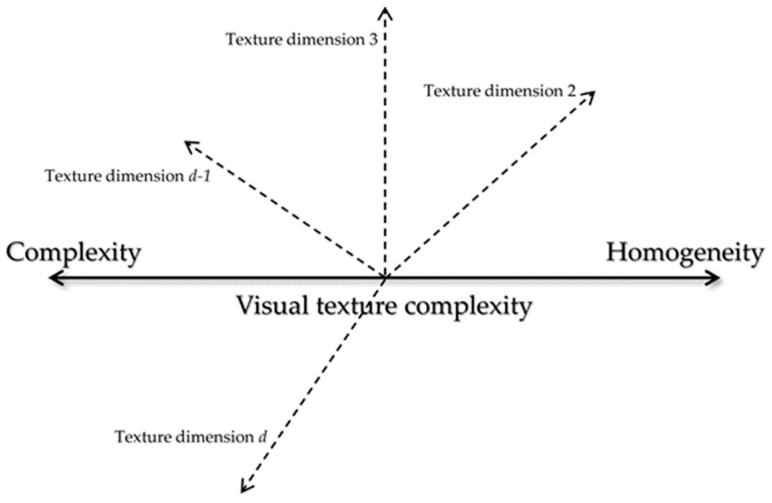
The hypothetical perceptual texture space with *visual texture complexity* as one of its dimensions.

Assuming that texture (e.g., color and gloss) is a multidimensional appearance attribute, the PCA results might suggest that *complexity* and *homogeneity* could be essentially the underlying attributes of the same visual texture dimension (described by PC1), with *complexity* at the negative extreme and *homogeneity* at the positive extreme of this dimension. We chose to call this dimension *visual texture complexity*. In other words, we can assume a *d*-dimensional hypothetical perceptual texture space with *visual texture complexity* as one of its dimensions. This dimension holds both *complexity* and *homogeneity* of texture. Figure 8 illustrates such a texture space. The PC1 values demonstrate that *color variation*, *randomness*, and *strongness* (with respective PC1 values −0.40, −0.37, −0.34) are strongly related to this dimension.

Considering the strong linear correlation between *VRS_H_* and *VRS_Cpx_* (see Figure 7), and the large-absolute-PC1 obtained for both *VRS_H_* and *VRS_Cpx_*, we could use either of the scores as the visual ratings of *visual texture complexity*. However, preliminary correlation analysis showed that unlike *VRS_Cpx_*, *VRS_H_* was inversely correlated with the computed texture measures. Therefore, we chose *VRS_Cpx_* as the visual ratings of *visual texture complexity* for further correlation analysis in this paper.

**Figure 9:**
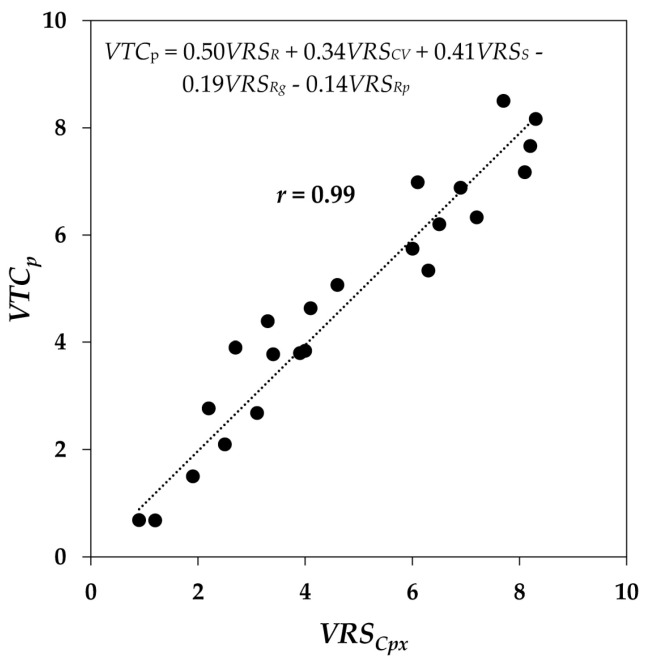
The correlation between *VRS_Cpx_* and *VTC_p_*. *VTC_p_* in Equation (2) is an intermediate variable defined to hold the ‘predicted’ *visual texture complexity* computed as the weighted sum of *VRS_R_*, *VRS_CV_*, *VRS_S_*, *VRS_Rg_*, and *VRS_Rp_* to have the best linear correlation with *VRS_Cpx_*.

To quantify the extent to which each attribute contributes to *visual texture complexity*, the multivariate regression method was used to model the correlation between *VRS_Cpx_* and the *VRS* of the rest of the attributes using the generic formula in Equation (2):

(2) $$VTC_{p}=k_{1}\left[VRS_{R}\right]+k_{2}\left[VRS_{CV}\right]+k_{3}\left[VRS_{S}\right]+k_{4}\left[VRS_{Rg}\right]+k_{5}\left[VRS_{Rp}\right]$$

where *VTC_P_* is an intermediate variable defined to hold the ‘predicted’ *visual texture complexity* values, computed as the weighted sum of *VRS_R_*, *VRS_CV_*, *VRS_S_*, *VRS_Rg_*, and *VRS_Rp_*, and *k_i_*; *i* = 1, 2, …, 5 are coefficients to express the contribution of each attribute to *VTC_P_*. Like *visual texture complexity*, *VTC_P_* is desired to be highly related to *VRS_Cpx_*. Therefore, the coefficients *k_i_* in Equation (2) were determined in such a way that the mean squared error (MSE) of the correlation between *VRS_Cpx_* and the corresponding computed *VTC_p_* values for the 23 textiles were minimized. The best linear correlation was achieved when *k*_1_, *k*_2_, *k*_3_, *k*_4_, and *k*_5_ were 0.50, 0.34, 0.41, −0.19, and −0.14, respectively. Figure 9 presents such a linear correlation between *VRS_Cpx_* and *VTC_p_*. These coefficients demonstrate that *randomness* of texture with *k*_1_ = 0.50, followed by *strongness* with *k*_3_ = 0.41 and *color variation* with *k*_2_ = 0.34 had the highest positive contributions to *VRS_Cpx_* and hence *visual texture complexity*: the higher these attributes are, the higher the *visual texture complexity* will be. On the other hand, greater *regularity* and *repetitiveness* contribute to lower *visual texture complexity*.

Given that texture is perceived as the frequency and spatial arrangement of color and/or luminance elements over the surface of a material [37], the *randomness*, *strongness*, *regularity*, and *repetitiveness* of texture could mainly be associated with the frequency and spatial distribution of such elements, regardless of their color, while *color variation* could be mainly associated with the frequency and diversity of color elements. These elements, also known as ‘texture primitives’ and ‘textons’ in the literature, are the smallest geometrical parts constituting a two-dimensional texture. Based on these correlations, *visual texture complexity* can be defined as the perceived degree of disorder or randomness in the distribution of color and luminance elements over the surface of a material, which depends on the size, shape, amount, and arrangement of such elements. We observed, during the visual assessment experiments, that a complex texture might be effortlessly discernable by the observer due to being salient and strong, but it might not necessarily be understandable, interpretable, or definable due to its random structure.

### 4.2. Correlation between Visual Texture Complexity and Image Descriptors

All image descriptors were linearly re-scaled to be within the same range as the VRS data (i.e., 0–10) for better visual comparison.

#### 4.2.1. sRGB Space

The R, G, and B channels in the sRGB space are highly correlated. This characteristic of the sRGB space makes it unsuitable for the individual evaluation of the effectiveness of each channel. Additionally, unlike the other color spaces investigated in this work, the luminance and chrominance information of sRGB images cannot be treated separately [71]. To obtain an approximation of luminance information in sRGB space, the sRGB images of the textiles were converted into the corresponding intensity images (see Appendix B).

**Figure 10:**
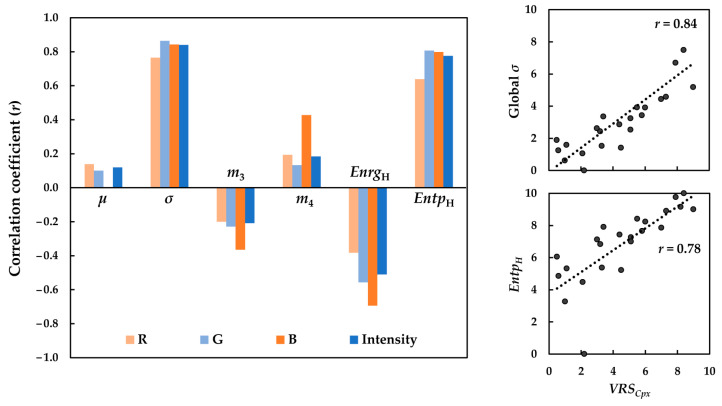
The correlation between the *VRS_Cpx_* of the textiles and various image descriptors computed for the R, G, and B channels and the sRGB intensity images of the textiles (**left**). Scatter plots of the global *σ* and *Entp*_H_ versus the corresponding *VRS_Cpx_* for the sRGB intensity images (**right**).

Global *µ* and *σ* as well as histogram-based *m*_3_, *m*_4_, *Enrg*_H_, and *Entp*_H_ were calculated separately for the R, G and B channels as well as the corresponding intensity images of the textiles, and re-scaled to be within the range 0–10. Correlations between *VRS_Cpx_* of the textiles and their respective computed image descriptors were then analyzed in terms of the correlation coefficient (*r*) for the 95% confidence interval (*p* < 0.5) separately for the R, G, and B channels and the intensity images. The bar graph in Figure 10 compares these correlation coefficients (*r*). The correlation coefficients (*r*) in Figure 10 showed that amongst all of the image descriptors, global *σ*, as a measure of image contrast, followed by *Entp*_H_ as an indicator of how random the distribution of image pixel values is, exhibited the highest positive correlations with *VRS_Cpx_*. *Enrg*_H_, as a measure of how few the number of pixel values in the image is, had a negative correlation with the visual data. On the other hand, there was no correlation between the global *µ* and the visual data. This proves that the mean is not a good candidate for quantifying the *visual texture complexity*, as expected. Central moments, *m*_3_ and *m*_4_, also exhibited weak correlations with the visual data.

The results in Figure 10 also revealed that each image channel tended to have a different performance in quantifying the *visual texture complexity* in a way that the highest correlations always belonged to the G channel, while the R channel always had the lowest correlations. This behavior might be attributed to the greater amount of useful information and less noise and blurriness provided by the G channel. Moreover, this could be due to the fact that the 23 image textures had a dominant blue-green color.

As mentioned previously, due to high cross-correlations between the R, G, and B channels in sRGB, comparing the image chromatic and achromatic/luminance components in terms of their contribution to *visual texture complexity* is not straightforward in the sRGB space. However, as can be seen in Figure 10, the image descriptors calculated for the intensity images of the textiles exhibited correlations that were fairly comparable with those of the best performing channel (i.e., the G channel). Scatter plots of the global *σ* and *Entp*_H_ of the sRGB intensity images against the corresponding *VRS_Cpx_* of the textiles are also depicted in Figure 10. Overall, the results indicate that the luminance content of the images might provide sufficient information for the objective quantification of texture *complexity*. Moreover, the choice of the image channel is a crucial step when working with color images in the sRGB space.

#### 4.2.2. Luminance–Chrominance Color Spaces

Transformations of the sRGB images in HSV, YC_b_C_r_, I_1_I_2_I_3_, and CIELAB (for CIE standard illuminant D65) color spaces were employed to compute the global image descriptors. The formulas for performing such transformations are given in Appendix B. Unlike the sRGB space, luminance–chrominance color spaces allow for the separate treatment of the color and luminance components of color images. V, Y, I_1_, and CIE L* are luminance channels, while H and S, C_b_, and C_r_, I_2_ and I_3_, and CIE a* and b* are the chrominance channels in the HSV, YC_b_C_r_, I_1_I_2_I_3_, and CIELAB color spaces, respectively. We must remind our readers that the term ‘chrominance’ here is different to that of ‘chroma’, as one of the three dimensions of color in the Munsell color system. Interested readers are referred to [64] for more information about the standardized color spaces available for image analysis.

**Figure 11:**
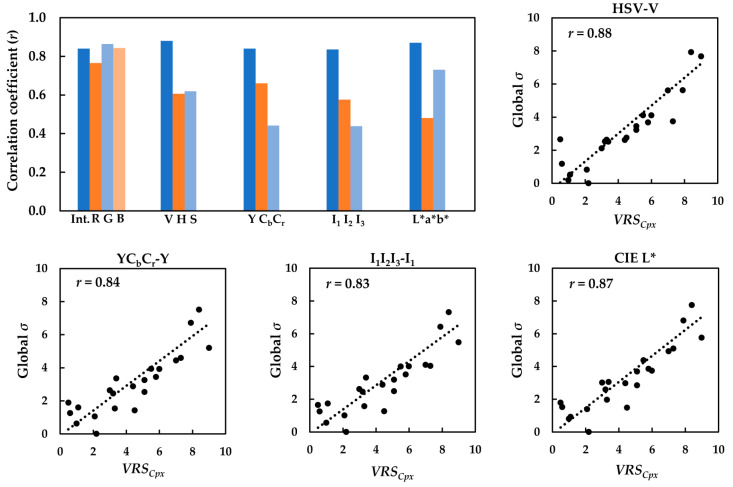
The correlation between the *VRS_Cpx_* of the textiles and global *σ* of the luminance and chrominance channels of the textile images in the sRGB, HSV, YC_b_C_r_, I_1_I_2_I_3_ and CIELAB color spaces. Here, ‘Int.’ refers to the sRGB intensity image (**top left**). Scatter plots of the global *σ* versus the corresponding *VRS_Cpx_* for the luminance channel of the textile images in the HSV, YC_b_C_r_, I_1_I_2_I_3_, and CIELAB color spaces.

Correlations between the global *µ* and *σ* of the luminance and chrominance channels of the images with the respective *VRS_Cpx_* were determined in terms of the correlation coefficient (*r*). Similar results were observed: the global *σ* performed significantly better than the global *µ* in terms of its relationship with the visual data. The bar graph in Figure 11 compares the correlation coefficients (*r*) of the relationships between the global *σ* of the images and *VRS_Cpx_* of the textiles, separately, for the luminance and chrominance channels in all five color spaces.

Analysis of the correlations in Figure 11 clearly showed that the global *σ* of the luminance channel gave rise to markedly higher correlation coefficients (*r*) compared to the chrominance channels. In other words, there seems to be a strong linear relationship between the standard deviation of the image luminance channel and its texture complexity, regardless of the color space used. Scatter plots of the best performing image descriptor (i.e., global *σ* computed for luminance channel of the images in HSV, YC_b_C_r_, I_1_I_2_I_3_ and CIELAB) against the corresponding *VRS_Cpx_* are also presented in Figure 11.

Although the correlation coefficients (*r*) obtained for the two chrominance channels of the images were lower, one always tends to result in a better relationship between the visual data and the computed texture feature. For instance, CIE b* resulted in a higher correlation coefficient (*r*) compared to CIE a*. Again, this could be attributed to the dominant blue-green color of the images.

### 4.3. Correlation between Visual Texture Complexity and Texture Features

All texture features were linearly re-scaled to be within the range of 0–10 for a better visual comparison with the visual data.

#### 4.3.1. Co-Occurrence Matrix (CoM) Texture Features

**Figure 12:**
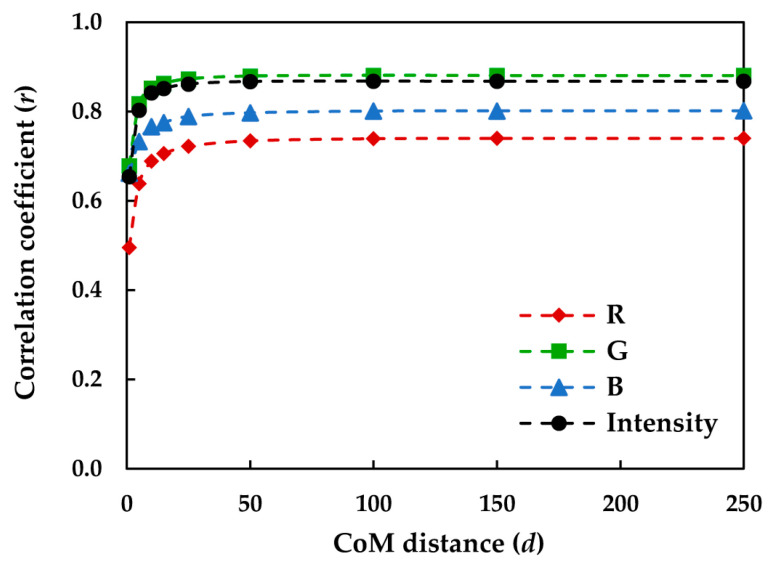
The correlation between the *VRS_Cpx_* of the textiles and *Entp_CoM_* of the textile images in the sRGB space improves by increasing the CoM distance (*d*) up to the certain value of *d* = 100 pixels, after which the correlation coefficient (*r*) remains almost unchanged.

Five rotation-invariant CoM features including *Enrg*_CoM_, *Cont*_CoM_, *Corr*_CoM_, *Entp*_CoM_, and *Homg*_CoM_ were computed for the textile images the in sRGB, HSV, YC_b_C_r_, I_1_I_2_I_3_, and CIELAB color spaces. The relationships between the *VRS_Cpx_* of the textiles and the corresponding CoM features of the textile images were subsequently determined. Preliminary analysis of the results demonstrated that the correlations depend on the CoM distance (*d*), in such a way that the correlation improves by increasing *d* up to the certain value of *d* = 100 pixels, after which the correlation coefficients (*r*) remain almost unchanged. Figure 12 shows such a dependency for *Entp_CoM_* of the R, G, and B channels and the sRGB intensity images.

Improving the correlations by increasing the CoM distance (*d*) most likely indicates that for this particular set of image textures, *Entp_CoM_* was able to detect the fundamental texture primitives of the images the best when the neighboring pixels were at least 100 pixels apart. This optimal CoM distance (*d*) is expected to comply with the observers’ field of view during the visual assessment of texture. The field of view is considered as the angle formed within the observer’s eyes with respect to the sample size, particularly, the size of the texture primitives observed on the physical sample. The scanning line of 100 pixels on the image corresponded to a line of 1.5 cm on the physical sample, forming the field of view of approximately 2° at the viewing distance of 40 cm.

**Figure 13:**
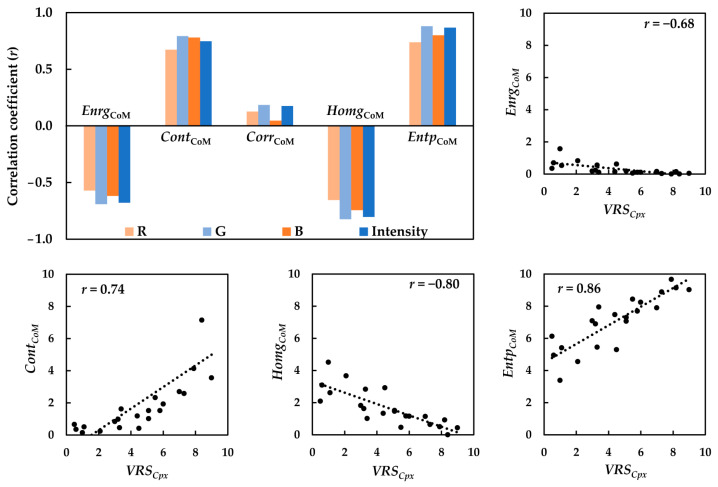
The correlation between the *VRS_Cpx_* of the textiles and the CoM features of the textile images computed with the CoM distance *d* = 100 pixels for the R, G, and B channels and the sRGB intensity images (**top left**). Scatter plots of *Enrg*_CoM_, *Cont*_CoM_, *Homg*_CoM_, and *Entp*_CoM_ versus the corresponding *VRS_Cpx_* for the sRGB intensity images.

The bar graph in Figure 13 compares the five CoM features computed with *d* = 100 pixels, in terms of their correlations with the visual data. Scatter diagrams of the CoM features with the highest correlation coefficients (*r*) (i.e., *Enrg_CoM_*, *Homg_CoM_ Cont_CoM_,* and *Entp_CoM_* versus the respective visual data) are also depicted in Figure 13. The correlation coefficients (*r*) in Figure 13 indicate that amongst all of the CoM features, *Entp*_CoM_, followed by *Cont*_CoM_, exhibited the highest positive correlations with *VRS_Cpx_*, with *Entp*_CoM_ performing slightly better than *Cont*_CoM_. *Homg*_CoM_ and *Enrg*_CoM_, on the other hand, had negative correlations with the visual data. Such relationships imply that a texture with higher entropy (or contrast) has lower homogeneity (or energy) and vice versa. Considering the mathematical expressions of these features, such inverse relationships were expected. There seems to be no relationship between *Corr*_CoM_ and *complexity*. Again, the highest correlations in the sRGB space belong to the G channel, followed by the sRGB intensity image, and the lowest correlations belong to the R channel.

**Figure 14:**
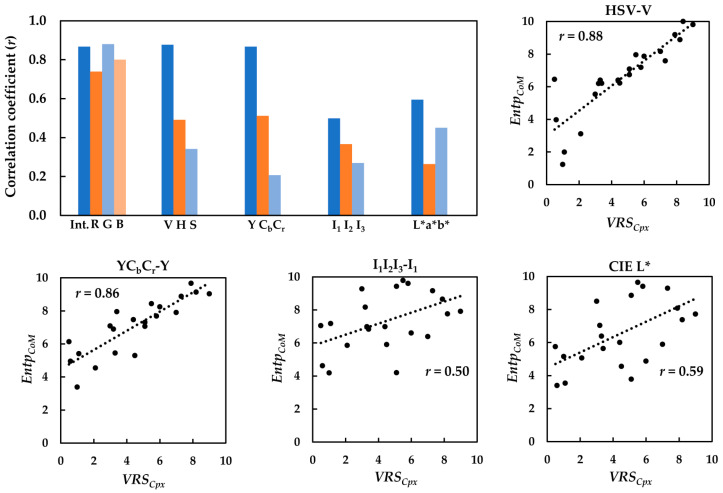
The correlation between the *VRS_Cpx_* of the textiles and *Entp*_CoM_ computed for the luminance and chrominance channels of the textile images in the sRGB, HSV, YC_b_C_r_, I_1_I_2_I_3_, and CIELAB color spaces with the CoM distance *d* = 100 pixels. Here, ‘Int.’ refers to the sRGB intensity image (**top left**). Scatter plots of *Entp*_CoM_ versus the corresponding *VRS_Cpx_* for the luminance channel of the textile images in the HSV, YC_b_C_r_, I_1_I_2_I_3_, and CIELAB color spaces.

The CoM features were also computed in the HSV, YC_b_C_r_, I_1_I_2_I_3_, and CIELAB color spaces, and their relationships with the *VRS_Cpx_* of the textiles were evaluated in terms of the correlation coefficient (*r*). Again, *Entp*_CoM_, followed by *Cont*_CoM_, gave rise to the highest positive correlations with the visual data. The correlation coefficients (*r*) computed for *Entp*_CoM_ of the luminance and chrominance channels of the textile images in the five color spaces were compared, as shown in the bar graph in Figure 14.

The correlation analysis in Figure 14 shows the markedly better performance of the luminance channel compared to the chrominance channels, regardless of the color space used. Figure 13 also presents the scatter diagrams of *Entp*_CoM_ of the images’ luminance channels in HSV, YC_b_C_r_, I_1_I_2_I_3_, and CIELAB against the respective visual data. The highest correlation coefficients (*r*) belonged to the V channel in the HSV, sRGB intensity image, and Y channel in YC_b_C_r_. On the other hand, the CIE L* and I_1_ poorly performed this time.

#### 4.3.2. Local Binary Pattern (LBP) Texture Features

As mentioned before, Ojala et al. [54] observed that uniform LBPs were associated with the most fundamental texture primitives present in the image such as bright points, dark points, edges, and corners. For instance, the first pattern in Figure 2b is expected to detect brighter points surrounded by a darker area. Similarly, the ninth pattern could detect darker points embedded in a brighter surrounding. The fourth and sixth patterns could detect corners, while the fifth pattern could detect edges in the texture. The remaining possible patterns were categorized as non-uniform ones.

For *N* = 8, the LBP histogram for each image texture had ten bins, resulting in a 10 × 1 feature vector, where elements of the vector were frequencies of the respective bins. The first nine bins were associated with the nine uniform LBP patterns (see Figure 2b), and the tenth bin was associated with all non-uniform patterns. The frequencies were linearly re-scaled to be within the range 0–10. We first analyzed the correlation between *VRS_Cpx_* and each element of the LBP feature vectors in the sRGB space. It was found that the correlation coefficients (*r*) obtained for the first nine bins were comparatively low, with the first bin having the lowest correlation, and no statistically significant difference between the correlation coefficients (*r*) of the remaining eight bins (i.e., bins 2–9). Based on these observations, the frequencies of the first nine bins, or the nine uniform LBPs, were determined and averaged for use as the single-number uniform LBP feature. Similarly, the frequency of the tenth bin was determined for use as the non-uniform LBP feature. We assumed that the non-uniform LBP feature could detect non-textured and homogeneous areas in the image texture.

**Figure 15:**
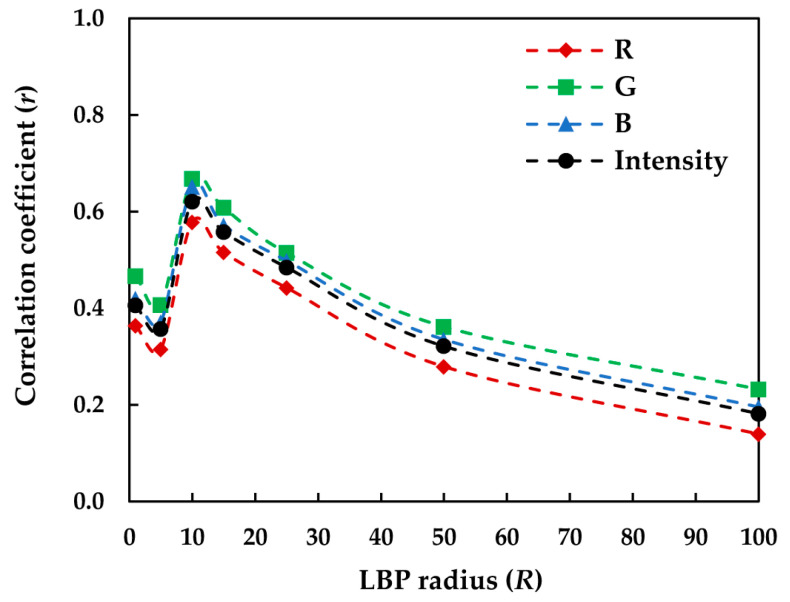
The correlation between the *VRS_Cpx_* of the textiles and rotation-invariant uniform LBP feature of the textile images in the sRGB space depends on the LBP radius *R*.

A preliminary analysis of the relationships between the *VRS_Cpx_* of the textiles and the corresponding LBP features in the sRGB space indicated that the correlations depended on the LBP radius *R* in such a way that the highest correlations for the uniform LBP feature were obtained when *R* = 10 pixels. Figure 15 illustrates such dependency for the R, G, and B channels and the sRGB intensity images of the textiles.

These results indicate that for this particular set of image textures, uniform LBPs were able to detect the texture primitives of the images at their best when they scanned the image over circular areas of approximately 20 pixels in diameter. A circular area of approximately 20 pixels in diameter on the image corresponded to a small circular area of approximately 3 mm in diameter on the physical textile sample, forming a field of view of approximately 0.5° at the viewing distance of 40 cm. The respective scanning line on the physical sample for *Entp*_CoM_ with the CoM distance *d* = 100 pixels was about 1.5 cm, forming the field of view of approximately 2° at the same viewing distance. This might suggest that uniform LBPs compute the texture in a more localized manner compared to the CoM features.

The high correlation coefficients (*r*) obtained for *Entp*_CoM_ suggests that a 2° field of view could reasonably represent the actual observer’s field of view during the visual assessment of texture. On the other hand, the low correlation coefficients (*r*) obtained for the LBP features indicate that the field of view of 0.5° might not accurately represent the actual observer’s field of view.

**Figure 16:**
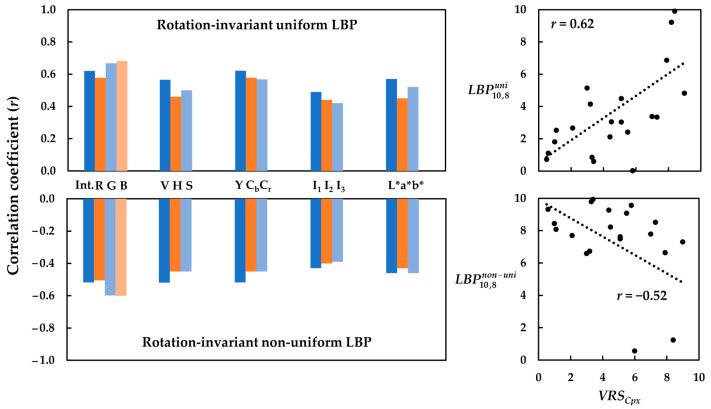
The correlation between the *VRS_Cpx_* of the textiles and uniform and non-uniform LBP features computed for the textile images with *R* = 10 pixels and *N* = 8 neighboring pixels in the sRGB, HSV, YC_b_C_r_, I_1_I_2_I_3_ and CIELAB color spaces. Here, ‘Int.’ refers to sRGB intensity image (**left**); Scatter plots of uniform and non-uniform LBP features versus the corresponding *VRS_Cpx_* for the sRGB intensity images (**right**).

Compared to the two previous texture measures (i.e., global *σ* and *Entp*_CoM_, which were successfully tested), the correlation coefficients obtained for the uniform and non-uniform LBP features were low. The bar graphs in Figure 16 compare such correlation coefficients (*r*) for the relationships between the *VRS_Cpx_* of the textiles and the corresponding rotation-invariant uniform and non-uniform LBP features with *R* = 10 pixels and *N* = 8 neighboring pixels, denoted here as $LBP_{10,8}^{uni}$ and $LBP_{10,8}^{non-uni}$, respectively.

Comparing the correlation coefficients (*r*) in Figure 16 revealed that uniform and non-uniform LBP features could clearly discriminate between the textile images with more prominent texture patterns and those with a more homogeneous structure. However, the overall correlations were low. Figure 16 also depicts the scatter plots of $LBP_{10,8}^{uni}$ and $LBP_{10,8}^{non-uni}$ versus the corresponding *VRS_Cpx_* of the textiles. $LBP_{10,8}^{uni}$—as the texture measure—had a positive correlation with *VRS_Cpx_*, while $LBP_{10,8}^{non-uni}$—as a potential measure for texture homogeneity—was inversely correlated with *VRS_Cpx_*. Again, the highest correlations belonged to the luminance channels of the images, irrespective of the color space used.

#### 4.3.3. Gabor Features

Rotation-invariant Gabor features (i.e., the mean and standard deviation of the energy of the filtered images, denoted as Gabor *µ* and Gabor *σ*, respectively) were computed for the luminance and color channels of the textile images in the sRGB, HSV, YC_b_C_r_, I_1_I_2_I_3_, and CIELAB color spaces. The relationships between the *VRS_Cpx_* of the textiles and Gabor features were then evaluated in terms of the correlation coefficient (*r*).

**Figure 17:**
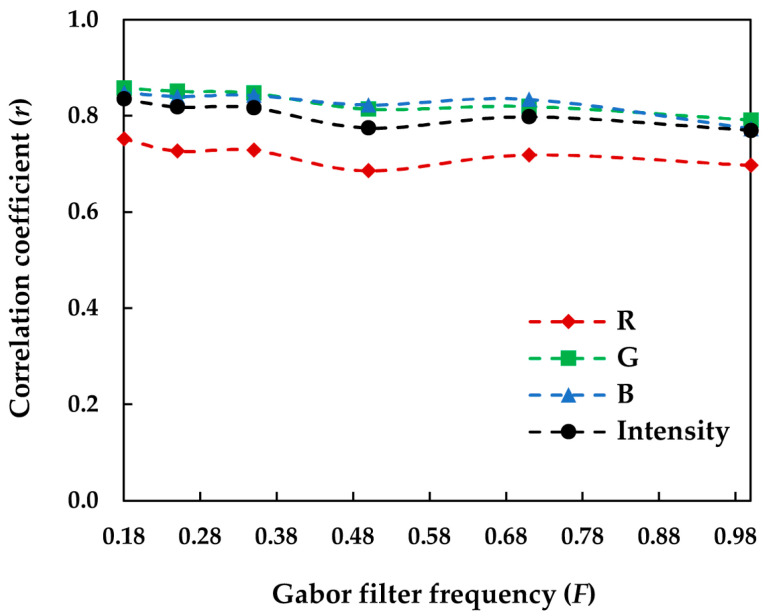
The correlation between the *VRS_Cpx_* of the textiles and rotation-invariant Gabor *σ* of the textile images in the sRGB space depends very slightly on the Gabor filter frequency (*F*).

To investigate the impact of Gabor filter frequency on such relationships, correlation coefficients (*r*) for various filter frequencies *F* were first compared in the sRGB space. It was found that the correlation coefficient (*r*) tended to generally decline by increasing the filter frequency *F* in such a way that the highest correlation was achieved for the smallest frequency used to design the Gabor filter bank (i.e., *F* = 0.18). However, the impact of the filter frequency on the results was quite subtle. Figure 17 illustrates the variation in the correlation coefficient (*r*) with the filter frequency in the sRGB space.

The very slight changes in the correlation coefficients (*r*) in Figure 17 indicate that the number of frequencies (*n_F_*) chosen to design the Gabor filter bank seems to have had little impact on image texture detection by the filter bank. A similar observation was made by Bianconi and Fernandez [34] for texture classification using Gabor filtering. They demonstrated that the number of frequencies and orientations used to design the filter bank did not significantly affect the accuracy of texture classification.

**Figure 18:**
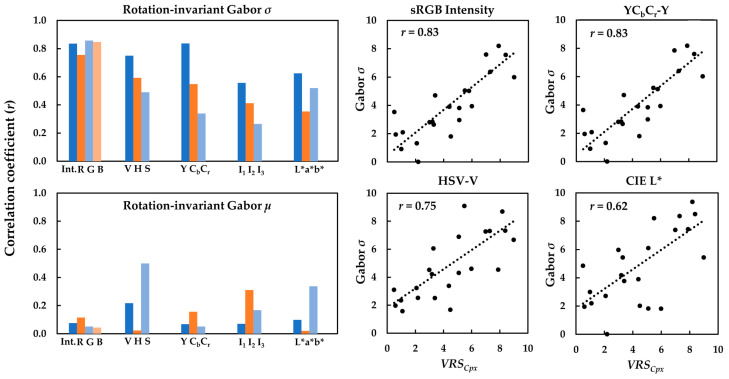
The correlation between the *VRS_Cpx_* of the textiles and the Gabor *σ* and Gabor *µ* computed for the luminance and chrominance channels of the textile images with the frequency *F* = 0.18 in the sRGB, HSV, YC_b_C_r_, I_1_I_2_I_3_, and CIELAB color spaces. Here, ‘Int.’ refers to the sRGB intensity image (**left**). Scatter plots of the Gabor *σ* versus the corresponding *VRS_Cpx_* for the luminance channel of the textile images in the sRGB HSV, YC_b_C_r_, and CIELAB color spaces (**right**).

Given the low impact of the filter frequency on the correlations, Gabor *µ* and Gabor *σ* of the textile images computed for *F* = 0.18 were used for further correlation analysis. The bar graphs in Figure 18 show the correlation coefficients (*r*) of the relationships between the *VRS_Cpx_* of the textiles and rotation-invariant Gabor *µ* and Gabor *σ* of the luminance and chrominance channels of the textile images in the sRGB, HSV, YC_b_C_r_, I_1_I_2_I_3_, and CIELAB color spaces.

A comparison of the correlation coefficients (*r*) in Figure 18 clearly showed the superiority of Gabor *σ* in quantifying the *visual texture complexity* of the textiles compared to Gabor *µ*. Again, the luminance channels of the images gave rise to stronger relationships between the visual data and computed Gabor *σ*, regardless of the color space used. Additionally, the highest correlation coefficients (*r*) belonged to the image luminance channels in sRGB and YC_b_C_r_, followed by HSV and CIELAB, while the poorest correlations were obtained in I_1_I_2_I_3_. Scatter plots of the Gabor *σ* computed for the luminance channel of the textile images against the corresponding *VRS_Cpx_* of the textiles in these color spaces are also depicted in Figure 18.

## 5. Discussion

Analyzing the results presented in the previous section helped us to address the three main objectives of this research introduced earlier in the Introduction:

### 5.1. Perception of Visual Texture Complexity

As mentioned briefly in the previous section, although the textile images employed in this study were color images, the poor relationships between the *VRS_Cpx_* of the textiles and the texture measures computed for the chrominance channels of the textile images demonstrated that the chrominance channels of the studied luminance–chrominance color spaces poorly performed in quantifying the *visual texture complexity*. For instance, even though the correlations obtained for the global *σ* of CIE b* were better than those of CIE a*, both CIE a* and b* performed worse than the luminance channel CIE L*. This indicates that the arrangement of the image texture elements that impacts the observer’s perception of *visual texture complexity* cannot be represented properly by the CIE a* and b* channels of the images. CIE L*, on the other hand, could represent it markedly better. This also applied to the HSV, YC_b_C_r_, and I_1_I_2_I_3_ color spaces. This must be carefully considered when choosing an image channel for quantifying the texture and texture complexity.

Moreover, the good performance of the image luminance channel in the five studied color spaces proved that variations in the luminance of the texture, or as one could call the *luminance contrast*, plays a crucial role in creating the so-called *visual texture complexity*. This finding is in agreement with the predictive model proposed in Section 4.1, where *visual texture complexity* of the textiles was found to be related to the spatial arrangement and spatial frequency of the texture elements, manifested as *randomness*, *strongness*, and *color variation*.

This supports the well-accepted fact that the human visual system interprets the visual signal as separate color and pattern information through separate neural pathways [72]. It also explains why joint color–texture features give rise to worse correlations with the visual data when compared to the correlations obtained for the pure color or pure luminance texture features. In other words, the human visual system is perfectly able to interpret achromatic texture, and color only enriches such interpretations. In this regard, it must be emphasized that typical textile materials usually exhibit lower levels of *visual texture complexity* compared to textures with a very high dynamic range of spatial frequency such as color fractals. Therefore, a thorough investigation using texture samples with a broad range of texture complexity and color contrast is needed to verify the present results.

### 5.2. Visual Texture Complexity and Its Correlation with Image Texture Measures

**Figure 19:**
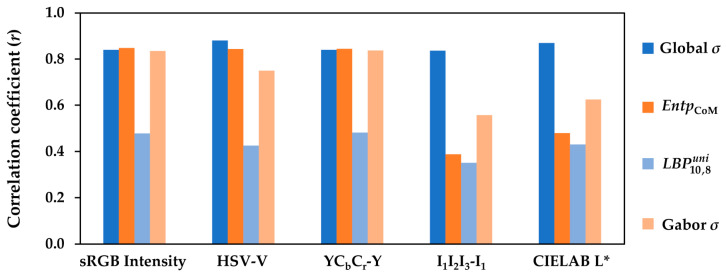
The performance of various texture measures computed for the luminance channel of the textile images in the sRGB, HSV, YC_b_C_r_, I_1_I_2_I_3_, and CIELAB color spaces in terms of their correlations with the *VRS_Cpx_* of the textiles given by a panel of observers.

A summary of the performance of the best performing texture measures from different classes investigated in this research (i.e., global *σ* as an image descriptor and *Entp*_CoM_, $LBP_{10,8}^{uni}$, and Gabor *σ* as texture features in quantifying the *visual texture complexity* of the textiles) is depicted in Figure 19.

The correlation coefficients (*r*) in Figure 19 were computed for the luminance channel of the textile images in the sRGB, HSV, YC_b_C_r_, I_1_I_2_I_3_, and CIELAB color spaces and exhibited the efficiency of each texture measure and color space in the objective quantification of the *visual texture complexity* of this particular set of textile samples.

As mentioned before, the global *σ* measures the spread in the image pixel values. A high global *σ* indicates an image with high contrast. The high correlation coefficients (*r*) obtained for the global *σ* in all five color spaces show that this simple, yet very informative image statistic could be a good candidate for deriving a quantitative measure for the *visual texture complexity*. Global *σ* performed the best when computed for the luminance channel of the images, where it provides a measure for the luminance contrast. The main drawback of this image descriptor as a texture measure is its sensitivity to changes in the illumination condition. However, it seems that global *σ* is much less dependent on the choice of color space compared to other texture features.

*Entp_CoM_* reflects the extent to which the pixel values are randomly distributed in the image. Although entropy itself is, by definition, spatially-invariant, the CoM entropy can be computed locally over a range of CoM distances, which makes it a more localized texture detector compared to global *σ*. *Entp_CoM_* exhibited the highest correlations with the *visual texture complexity* of the textiles in sRGB and YC_b_C_r_, with lower correlations in the I_1_I_2_I_3_ and CIELAB color spaces. This indicates that CoM features are more sensitive to changes in the image pixel values and are highly dependent on the choice of color space.

Although uniform and non-uniform LBP features are able to discriminate between the textured and non-textured images, we observed no strong correlation between these features and *visual texture complexity* of the textiles in this research. This might demonstrate that uniform LBP features are unable to provide sufficient information about the luminance contrast of these image textures.

Gabor filtering is a very powerful tool for a controlled localized extraction of texture information from images by tuning the filter parameters. Some evidence has been provided by the human vision research proving that human brain processes images by a frequency analysis routine [73]. It has also been found that two-dimensional Gabor functions fit the receptive fields of simple cells in the primary visual cortex of mammals [74]. The high correlations between the visual data and the corresponding Gabor *σ* of the textile images in the present study support such evidence. The correlation coefficients (*r*) were very close to those of the *Entp_CoM_* and global *σ* in the sRGB and YC_b_C_r_ color spaces. Moreover, Gabor *σ* performed better than *Entp_CoM_* in the I_1_I_2_I_3_ and CIELAB color spaces. Like *Entp_CoM_*, Gabor *σ* is sensitive to changes in the image pixel values and hence dependent on the choice of color space.

Overall, the results of the correlation analysis demonstrate that a robust texture measure for deriving a quantitative metric for *visual texture complexity* is one that is able to efficiently detect and quantify the luminance contrast of the texture.

To investigate the joint contribution of the four texture measures to *visual texture complexity*, a similar generic formula to Equation (2) was used to model the correlation between the *VRS_Cpx_* and the four computed texture measures for the sRGB intensity images:

(3) $$VTC_{p}=k_{1}\left[Global\;\sigma \right]+k_{2}\left[Entp_{CoM}\right]+k_{3}\left[LBP_{10,8}^{uni}\right]+k_{4}\left[Gabor\;\sigma \right]$$

where *VTC_p_* is again an intermediate variable to hold the predicted *visual texture complexity* values, and *k_i_*, *i* = 1, 2, …, 4 are coefficients to express the contribution of each texture measure to *VTC_p_*. By minimizing the mean squared error (MSE), the best correlation between *VRS_Cpx_* and *VTC_p_* was achieved when *k*_1_, *k*_2_, *k*_3_, and *k*_4_ were 0.39, 0.28, 0.03, and 0.30, respectively. These coefficients verify the results of the bivariate correlation analysis in terms of the effectiveness of the global *σ*, followed by Gabor *σ* and *Entp*_CoM_ in quantifying the *visual texture complexity*.

Considering the higher contributions of global *σ* and Gabor *σ* over the others, we performed an exploratory factor analysis [70] on the four texture measures for the sRGB intensity images to evaluate their possible inter-dependencies. The factor loadings/coefficients of the global *σ*, *Entp*_CoM_, $LBP_{10,8}^{uni}$, and Gabor *σ* for a single common factor analysis, together with their respective specific variances, are presented in Table 2.

The factor loadings in Table 2 show that the single common factor put the most weight on the global *σ* and Gabor *σ*. Their corresponding close to zero specific variances indicated that the two measures were almost entirely determined by this common factor. On the other hand, lower weights of $LBP_{10,8}^{uni}$ and *Entp*_CoM_ (and their much higher specific variances) indicated that these two measures were most likely described by other factors. In other words, the global *σ* and Gabor *σ* of the image textures in the sRGB space seem to be highly inter-dependent. This explains why both measures showed high correlations with the visual data in the sRGB and YC_b_C_r_ spaces.

### 5.3. Most Suitable Color Spaces for Quantitative Evaluation of Visual Texture Complexity

A comparison between the correlation coefficients (*r*) obtained for various color spaces in Figure 19 revealed that sRGB and YC_b_C_r_, followed by HSV, outperformed the I_1_I_2_I_3_ and CIELAB color spaces. YC_b_C_r_ and HSV were immediate transformations of sRGB: for each image pixel, *Y* in YC_b_C_r_ is the weighted sum of the *R*, *G*, and *B* values, and *V* in HSV is the maximum of the *R*, *G*, and *B* values. Therefore, it appears that for this particular set of image textures, when quantifying the luminance contrast matters, the sRGB space and its transformations (except for I_1_I_2_I_3_) seemed to be more reliable than the uniform color spaces such as CIELAB. I_1_I_2_I_3_ exhibited the worst performance amongst all.

It must be emphasized that although the correlation coefficients (*r*) obtained for the two chrominance channels of the images were always lower than those of the luminance channel for all five color spaces, one always tended to outperform the others in terms of correlation with the visual data. The best performing chrominance channels were G in sRGB, H in HSV, C_b_ in YC_b_C_r_, I_2_ in I_1_I_2_I_3_, and b* in CIELAB. This could also be attributed to the dominant blue-green color of the images. In other words, the selection of a suitable color space and color channel for texture quantification tasks is highly influenced by the color characteristics of the image textures.

## 6. Conclusions

The aim of this research was to investigate how observers interpret the texture complexity of a series of textile fabrics, and the possibility of finding a texture metric to quantify this attribute. The results of the visual assessment experiments revealed that the observers tended to evaluate the texture of the textiles by the aid of several visual cues: *homogeneity*, *randomness*, *repetitiveness*, *regularity*, *color variation*, and *strongness*, and they used these cues to interpret the *complexity* of texture as a higher-level attribute. We chose to call this attribute the *visual texture complexity*. Based on the results of the correlation analysis, we could generally define *visual texture complexity* as the perceived degree of disorder or randomness in the distribution of the color and luminance elements over the surface of a material, which depends on the size, shape, amount, and arrangement of such elements.

To investigate whether there is any potential quantitative metric for *visual texture complexity*, images of the textiles in the sRGB, HSV, YC_b_C_r_, I_1_I_2_I_3_ and CIELAB color spaces were used to compute a number of image statistics and texture features. First-order image statistics, together with co-occurrence matrix, local binary pattern, and Gabor filtering, were chosen as some of the most popular image and texture operators for this purpose. Linear relationships between the ratings of *visual texture complexity* given by the observers through rank ordering the textiles and the computed features of the textile images were analyzed. Amongst the several first-order image statistics, the standard deviation of the luminance channel of the images showed the highest correlations with the visual data in sRGB, HSV, YC_b_C_r_, I_1_I_2_I_3_ and CIELAB color spaces. Additionally, entropy of the co-occurrence matrix and the standard deviation of the image energy after convolving with the Gabor filter, both computed for luminance images in sRGB and YC_b_C_r_, showed the highest correlations with the visual data. Such correlations for the uniform LBP features were comparatively low.

The results demonstrate that features that are able to efficiently quantify the image luminance contrast exhibit better correlations with *visual texture complexity*. The highest correlations were always obtained for the luminance channel of the images, indicating the arrangement of the image texture elements, which impacts the observer’s perception of *visual texture complexity*, cannot be represented properly by the chrominance channels, particularly for the luminance–chrominance color spaces. Moreover, among the two chrominance channels of the images, one always tended to outperform the other in terms of the correlation with the visual data. This suggests that the choice of a suitable color space and color channel for image texture computation is highly influenced by the color characteristics of the texture database. However, it must be emphasized that these results are strongly associated with the characteristics of the texture database used and might be limited to the tested sample set and experimental conditions. The selected sample set was limited to textile materials that usually exhibit lower levels of visual complexity. Moreover, the number of samples and their texture diversity were limited. Additionally, sRGB and its transformations are considered as device dependent color spaces. Hence, changing the image acquisition device and/or illumination setup could result in different image representations, and therefore different correlations with the visual data. Further research using a more versatile texture database is needed to verify the present results. One could suggest the use of an image-based sample set (instead of physical samples) to verify the findings of the present research.

## Figures and Tables

**Table 1:** The results of the PCA on the *VRS* of the seven visual texture attributes: the first two principal component coefficients, PC1 and PC2.

Variable	PC1	PC2
*VRS_H_*	0.44	−0.04
*VRS_R_*	−0.37	−0.37
*VRS_Rp_*	−0.08	0.47
*VRS_Rg_*	0.16	0.46
*VRS_CV_*	−0.40	0.11
*VRS_S_*	−0.34	0.05
*VRS_Cpx_*	−0.42	−0.15

**Table 2:** The results of the factor analysis on the computed global *σ*, *Entp*_CoM_, $LBP_{10,8}^{uni}$, and Gabor *σ* of the sRGB intensity images of the textiles.

Texture Measure	Factor Loading	Specific Variance
Global *σ*	1.00	0.01
*Entp*_CoM_	0.83	0.32
$LBP_{10,8}^{uni}$	0.64	0.60
Gabor *σ*	0.96	0.08

## Data Availability

HyTexiLa, the dataset of hyperspectral reflectance images can be downloaded from https://color.univ-lille.fr/datasets (accessed on 7 September 2022).

## Appendix A. The Written Instruction about the Visual Assessment Experiment

**Introduction to Psychophysical Experiment:**

This psychophysical experiment is to investigate how human observers perceive the texture of textile materials. According to the International Commission on Illumination, CIE, the appearance of an object can be subdivided into at least four attributes, namely color, gloss, translucency, and texture. This experiment only focuses on texture. To understand better, imagine that you have an old sofa at home. The fabric of the seats is now worn out because of the friction for so many years. You decide to replace the fabric with a new one, so you pick up the phone and call the nearest upholstery fabric producer to see if they have it available in their product line. How would you describe the texture of the fabric to the salesperson in the simplest way so that they can find it for you?

In a previous experiment, we found that observers tend to describe the texture of textiles using the following words/qualities:

- Homogeneity
- Randomness
- Repetitiveness
- Regularity
- Color variation
- Strongness
- Complexity

In this experiment, we are interested in your understanding of these qualities, and would like to ask you to perform a sorting task on a series of fabrics with respect to these qualities. If you need to check the meaning of any of these words, you can use online dictionaries and then use your own interpretation to perform the experiment.

**How to perform the experiment:**

This is a rank ordering experiment. A rank ordering experiment is essentially a sorting task. There is a set of 23 textile fabrics. The experimenter will ask you to sit at the table and then present you with all the fabrics. You will have 5 min to familiarize yourself with the samples and evaluate them in terms of their texture. The experimenter will then randomly pick one of the above texture qualities and ask you to sort the fabrics on the table based on how much they represent that quality, from the lowest to the highest. To do the task, you can ask yourself questions like these:

- How homogeneous is fabric #1?
- Is there any fabric with higher homogeneity than fabric #1?

After you have finished the sorting, the experimenter will pick the next texture quality and ask you to repeat the sorting. Please avoid leaning forward or backward while sorting to keep your distance from the table.

We deeply thank you for participating in the experiment!

## Appendix B. Formulas for Transforming sRGB to HSV, YC_b_C_r_, Ohta’s I_1_I_2_I_3_ and CIELAB Color Spaces

Formulas used in this paper to transform the colors in the sRGB space to the corresponding colors in the HSV, YC_b_C_r_, Ohta’s I_1_I_2_I_3_ and CIELAB spaces are as follows. In these formulas, *R*, *G*, and *B* denote the red, green, and blue pixel values of the sRGB image, respectively. For an 8-bit sRGB image, *R*, *G*, and *B* are within the range 0–255.

### Appendix B.1. sRGB to Intensity Image

Intensity image (I), also known as the grayscale image, is simply the linear transformation of the sRGB image using the weighted sum of the R, G, and B values. The following equation was used to compute the intensity image (I) from the sRGB image [71]:

(A1) I=0.2989R+0.5870G+0.1140B

### Appendix B.2. sRGB to HSV Color Space

The HSV color space defines colors in terms of three components: hue angle, saturation, and value. Such color components are believed to relate well to human color perception. The hue angle (*H*) represents a true color such as red, yellow, purple, etc. Saturation (*S*) and value (*V*) represent the purity and brightness of the color, respectively. The *H*, *S*, and *V* components of the sRGB image is calculated using the below equations [75]:

(A2a) $$H=\left\{ \begin{array}{ll} 0 & if_{}^{}\;T=0\\ 60{\left(\frac{G-B}{255T}\mathrm{mod}6\right)}_{}^{} & if_{}^{}\;T_{\max}=R/255\\ 60{\left(\frac{B-R}{255T}+2\right)}_{}^{} & if_{}^{}\;T_{\max}=G/255\\ 60{\left(\frac{R-G}{255T}+4\right)}_{}^{} & if_{}^{}\;T_{\max}=B/255 \end{array}\right.$$

(A2b) $$S=\left\{ \begin{array}{ll} 0 & if_{}^{}\;T_{\max}=0\\ T/T_{\max} & if_{}^{}\;T_{\max}\neq 0 \end{array}\right.$$

(A2c) $$V=T_{\max}$$

With

(A2d) $$T_{\max}=\max(R,G,B)/255\;T_{\min}=\min(R,G,B)/255\;T=T_{\max}-T_{\min}$$

### Appendix B.3. sRGB to YCbCr Color Space

The YC_b_C_r_ color space is a widely used standardized color model in digital video processing. It separates the luminance and chrominance components of colors into the luminance component (*Y*) and the chrominance components blue (*C_b_*) and red (*C_r_*). sRGB images can be transferred into the YC_b_C_r_ color space using the following linear transformations [71]:

(A3) $$\left[\begin{array}{l} Y\\ C_{b}\\ C_{r} \end{array}\right]=\left[\begin{array}{l} 16\\ 128\\ 128 \end{array}\right]+\left[\begin{array}{lll} 65.481_{}^{} & 128.553_{}^{} & 24.966\\ -37.797 & -74.203 & 112\\ 112 & -93.786 & -18.214 \end{array}\right]\left[\begin{array}{l} R/255\\ G/255\\ B/255 \end{array}\right]$$

### Appendix B.4. sRGB to Ohta’s I1I2I3 Color Space

The 1_1_1_2_1_3_ color space, proposed by Ohta et al. [76] for color region segmentation, represents colors in terms of three color features *I_1_*, *I_2_*, and *I_3_*, where *I_1_* is the luminance, and *I_2_* and *I_3_* are the chrominance components of the color. The three features can be computed using the following equations [71]:

(A4a) $$I_{1}=\frac{R+G+B}{3}$$

(A4b)
$$I_{2}=\frac{R-B}{2}$$

(A4c)
$$I_{3}=\frac{2G-R-B}{4}$$

### Appendix B.5. sRGB to CLELAB Color Space

The CLELAB color space recommended by the international lighting commission CIE in 1976, is a perceptually uniform color space in which color differences perceived by the human visual system correlate well with the Euclidian differences in the color coordinates *L**, *a**, and *b**. CIE *L** defines the lightness, and CIE *a** and *b** represent the redness–greenness, and yellowness–blueness of the color, respectively. In order to transform sRGB images to the CIELAB color space, the *R*, *G*, and *B* values were first converted to intermediate CIEXYZ tristimulus values using the following linear transformations [71]:

(A5a) $$\left[\begin{array}{l} X\\ Y\\ Z \end{array}\right]=\left[\begin{array}{l} 0.412453_{}^{}{{}_{}^{}}_{}^{}{{}_{}^{}}_{}^{}\;\;\;0.357580_{}^{}{{}_{}^{}}_{}^{}{{}_{}^{}}_{}^{}\;\;\;0.180423\\ 0.212671_{}^{}{{}_{}^{}}_{}^{}{{}_{}^{}}_{}^{}\;\;\;0.715160_{}^{}{{}_{}^{}}_{}^{}{{}_{}^{}}_{}^{}\;\;\;0.072169\\ 0.019334_{}^{}{{}_{}^{}}_{}^{}{{}_{}^{}}_{}^{}\;\;\;0.119193_{}^{}{{}_{}^{}}_{}^{}{{}_{}^{}}_{}^{}\;\;\;0.950227 \end{array}\right]\left[\begin{array}{l} R\\ G\\ B \end{array}\right]$$

The CIE *L**, *a**, and *b** color coordinates under a particular illuminant with the tristimulus values *X_n_Y_n_Z_n_* are subsequently calculated using the following equations:

(A5b) $$L*=116f\left(\frac{Y}{Y_{n}}\right)-16$$

(A5c) $$a*=500\left[f\left(\frac{X}{X_{n}}\right)-f\left(\frac{Y}{Y_{n}}\right)\right]$$

(A5d) $$b*=200\left[f\left(\frac{Y}{Y_{n}}\right)-f\left(\frac{Z}{Z_{n}}\right)\right]$$

With

(A5e) $$f\left(U\right)=\left\{ \begin{array}{ll} U^{\frac{1}{3}}{{}_{}^{}}_{}^{}{{}_{}^{}}_{}^{}{{}_{}^{}}_{}^{}{{}_{}^{}}_{}^{}{{}_{}^{}}_{}^{}{{}_{}^{}}_{}^{}{{}_{}^{}}_{}^{}{{}_{}^{}}_{}^{}{{}_{}^{}}_{}^{}{{}_{}^{}}_{}^{} & if_{}^{}{}_{}^{}\;U>0.008856\\ 7.787U+{\frac{16}{116}}_{}^{}{{}_{}^{}}_{}^{}{{}_{}^{}}_{}^{}{}_{}^{} & if_{}^{}{}_{}^{}\;U\leq 0.008856 \end{array}\right.$$
